# Incidence and predictors of recovery from anaemia within an HIV-infected South African Cohort, 2004-2010

**DOI:** 10.11604/pamj.2014.19.114.3600

**Published:** 2014-10-01

**Authors:** Zibusiso Ndlovu, Tobias Chirwa, Simbarashe Takuva

**Affiliations:** 1 School of Public Health, Faculty of Health Sciences, University of the Witwatersrand, Johannesburg, South Africa; 2 Clinical HIV Research Unit, Department of Internal Medicine, School of Clinical Medicine, Faculty of Health Science, University of the Witwatersrand, Johannesburg, South Africa

**Keywords:** Haemoglobin, human immunodeficiency virus, AIDS, Themba Lethu clinical cohort, antiretroviral therapy

## Abstract

**Introduction:**

Anaemia is one of the most frequent haematological complications in HIV-infected persons. Understandingfactors associated with recovery from anaemia during ART is vital in improving clinical outcomes since anaemia is a strong predictor of mortality.

**Methods:**

Cohort study of 12,441 HIV-infected adults initiating ART between 2004-2010 in Johannesburg, South Africa. A further 2,489 patients with prevalent anaemia at ART initiation were examined to determine the incidence and predictors of recovery from anaemia. Cox proportional hazards models were fitted to investigate predictors of recovery from anaemia.

**Results:**

Of the 2,489 patients with prevalent anaemia, most patients (n = 2,225, 89.4%) recovered from anaemia. Median time to anaemia recoverywas 3.9 months (IQR: 3.22-6.20) and incidence rate was 180 per 100person years (95% CI: 172-187). In univariateanalysis, sex, CD4 count, BMI, WHO stage, employment status, smoking status and presence of tuberculosis at initiation ofART were significant predictors of recovery from anaemia. However in multivariateanalysis, predictors of recovery from anaemia were: male sex-HR: 1.43 (95% CI: 1.29-1.59) p< 0.001, advanced WHO stage III/IV - HR: 1.17 (95% CI: 1.07-1.29) p = 0.001). There was no significant association with CD4 count in multivariate analysis.

**Conclusion:**

A large proportion of HIV infected patients with anaemia at baseline recover early during the course of ART. Females and those with less advanced WHO stage seem to be at higher risk of poor recovery from anaemia. Understanding the predictors for poor recovery from anaemia would allow closer follow-up and more targeted interventions thus reducing excess anaemia and mortality burden.

## Introduction

Anaemia is one of the most frequent haematological complications seen in people with human immuno-deficiency virus (HIV) and acquired immuno-deficiency syndrome (AIDS) [1, 2]. Among HIV infected individuals, the prevalence of anaemia at initiation of antiretroviral therapy (ART) is reported to range between20% and90% in different clinical settings [3–5]. Anaemia in HIV infected individuals has multifactorial aetiologies which complicate its differential diagnosis and treatment.

It has been demonstrated that haemoglobin levels provide prognostic information independent of that provided by the CD4 lymphocyte count and HIV viral load [6]and the presence of anaemia has been shown to be a significant predictor of progression to AIDS, and is independently associated with an increased risk of death [4, 6–11]. Conversely, recovery from anaemia among HIV-infected patients has been shown to be associated with decreased risk of disease progression to approximately the same level as in patients who have never had anaemia [12]. In addition, recovery from anaemia is associated with improved survival [13, 14]. Although the burden of anaemia amongHIV infected patients has reducedsince the introduction of ART [1, 4], anaemia remains prevalent even among patients on ART, with approximately 18%-46% of patients, anaemic one year after initiating ART[10] and it continues to be a common contributor of morbidity and mortality [8].

Better understanding of the predictors of recovery from anaemia will allow closer follow-up and more targeted interventions among patients initiating ART with anaemia thus help to improve morbidity and mortalityoutcomes. Whilst numerous studies have identifiedpredictors of persistent anaemia [15–18] and the most common include race, gender, CD4 cell count less than 200 cells/mm^3^, HIV viral load, MCV, presence of opportunistic infections, zidovudine use and WHO clinical staging, there is scanty information in the literature on predictors of recovery from anaemia, more-soreinlimited-resource settings. In limited-resource settings, understanding the factors influencing recovery from anaemia is even more crucial since this setting bears a disproportionate burden of anaemia due to prevalent co-morbidities like malnutrition, tuberculosis, malaria and other parasitic infections.

In this study, we set to examine the incidence of recovery from anaemia among patients initiating ART in a large HIV-infected clinical cohort in Johannesburg, South Africa. Also, we set to determine predictors associated with recovery from anaemia during the follow-up period.

## Methods

### Study population and setting

The Themba Lethu HIV Clinic in Johannesburg, South Africa is one of the largest HIV care and treatment clinics in South Africa with over 21,000 patients initiated onto ART since 2004. Details of this cohort have been published previously [19].

### Study cohort eligibility criteria

We conducted a prospective cohort analysis of HIV infected ART naïve patients >18 years of age, who initiated standard first line ART at Themba Lethu Clinic (TLC) between April 2004 and June 2010 with CD4 counts =350 cells/ml. Pregnant women, women in the post-partum period (within 6 weeks post-delivery) and patients on agents which enhance haematopoiesis (e.g. iron supplements, transfusion etc.) were excluded from the study. At the time, standard public-sector ART in South Africa consisted of stavudine (d4T) or zidovudine (AZT) with lamivudine (3TC) and either efavirenz (EFV) or nevirapine (NVP) [20]. We restricted the analysis to patients who had baseline haemoglobin done and 2 subsequent haemoglobin measurements and the final analytic cohort consisted of patients who had anaemia at baseline (haemoglobin level < 10g/dl). Baseline haemoglobin was defined as the latest value 6 months before to 7 days after ART initiation. At TLC patients are scheduled to have a full blood count at ART initiation, four months after initiation and then six monthly thereafter. If initiated onto AZT containing regimen, a full blood count is done at initiation, monthly for the first three months on treatment and then six monthly thereafter. Only patients with a contraindication to d4T at ART initiation would be given AZT in place of d4T (i.e. pregnant women or patients receiving tuberculosis treatment). AZT is contraindicated in patients with haemoglobin < 10g/dl [20].

### Variable definitions

Anaemia was defined according to the WHO toxicity grading systems for anaemia as haemoglobin less than 10 g/dl [17]. In addition, haemoglobin less than 10 g/dl was chosen so that our study is comparable with most local studies [19]. Recovery from anaemia was defined as the first time point at which there was resolution of low haemoglobin (<10 g/dl) to within normal levels (=10 g/dl) during the study follow up period.

For the analysis, person-time accrued from treatment initiation until the earliest of: (1) recovery from anaemia; (2) death; (3) loss to follow-up (defined as 4 months late for last scheduled clinic visit); (4) transfer; or (5) close of the dataset on 30^th^June 2012.

### Statistical analysis

All data analyses were conducted in STATA version 12 (Stata Corp., College Station, Texas, US). Using the Log-rank test for sample size and power estimation, assuming an alpha of 5% and assuming that more than 70% of the patients would recover from anaemia and an effect size hazard ratio of 1.5, our sample size of 2,500 was highly powered (power > 90%). We computed period incidence rates and cumulative probabilities of recovery from anaemia at initiation. Cox proportional hazard models were fitted to investigate associations between baseline characteristics and recovery from anaemia. Variables that had a p-value < 0.25 on univariate analysis were considered as potential candidates for inclusion into the multivariatemodels. We then employed the stepwise forwardselection method to arrive at the final multivariate model. The following explanatory variables available at baseline were considered for inclusion into multivariate models; gender, age, employment status, education status, smoking status, alcohol consumption status, body mass index (BMI), WHO clinical staging, tuberculosis status and baseline CD4 count. CD4 count was forced into models as a priori. The proportionality assumption was checked (using log (-log (survival)) over time for each covariate) and was not violated. Overall goodness of fit of the final model was assessed using Cox-Snell residuals and there was good fit. In order to understand the extent to which loss to follow up or death may have biased our estimates, we furtherextended our analysis to compare the baseline characteristics of patients who were lost to follow up or died versus those who completed follow-up for the duration of the study. The Pearson's chi-square test and the u-Mann-Whitney tests were used to compare categorical and continuous variables respectively in this extended analysis.

Permission to conduct the study was granted by the Human Research Ethics Committee of the University Of Witwatersrand and use of Themba Lethu Clinic data was approved by the superintendent of Helen Joseph Hospital.

## Results

### Characteristics of patients with anaemia at initiation of ART

Between April 2004 and June 2010, 12,441 HIV positive subjects initiating ART at the TLC had baseline haemoglobin measurements. Among these, 26.5% (n = 3,299) had haemoglobin level < 10g/dl. We excluded 45 patients less than 18years of age and 765 patients who either did not return for follow-up or had no subsequent haemoglobin measurements. This left 2,489 patients with anaemia for analysis. The mean age of patients was 36.7 years (s.d. 8.6), mean haemoglobin was 8.7g/dl (s.d. 1.1) and the majority of patients were female, 73.5% (n = 1,830). Most patients 89.5% (n = 2,043) were initiated on stavudine/lamivudine/efavirenz regimen while < 2% were initiated ona zidovudine containing regimen. This cohort was immunosuppressed at initiation of ART, mean CD4 count was 83 cells/ml (s.d. 69) and 53.1% (n = 1,322) had advanced WHO stage (WHO stage III/IV). See Table 1. The prevalence and risk factors associated with anaemia for this cohort have previously been reported [19, 21].

**Table 1:** Association between periconceptional folic acid intake and sociodemographic/maternal obstetric characteristics of the participants

Periconceptional folic acid					
Variable	Taken No (%)	Not taken No (%)	Total No (%)	χ^2^	p value
**Age (years)**					
< 30	72 (27.0)	195 (73.0)	267 (100.0)	1.190	0.275
≥ 30	91 (31.2)	201 (68.8)	292 (100.0)		
Total	163 (29.2)	396 (70.8)	559 (100.0)		
**Marital status**					
Single	5 (33.3)	10 (66.7)	15 (100.0)	0.130	0.718
Married	158 (29.0)	386 (71.0)	544 (100.0)		
Total	163 (29.2)	396 (70.8)	559 (100.0)		
**Educational status**					
University education	74 (53.6)	64 (46.4)	138 (100.0)	53.089	<0.001*
Others	89 (21.1)	332 (78.9)	421 (100.0)		
Total	163 (29.2)	396 (70.8)	559 (100.0)		
**Socioeconomic class**					
Managerial/Professional	48 (55.8)	38 (44.2)	86 (100.0)	41.194	<0.001*
Intermediate class	38 (33.6)	75 (66.4)	113 (100.0)		
Working class	77 (21.4)	283 (78.6)	360 (100.0)		
Total	163 (29.2)	396 (70.8)	559 (100.0)		
**Gravidity**					
Primigravida	75 (41.0)	108 (59.0)	183 (100.0)	18.415	<0.001*
Multigravida	88 (23.4)	288 (76.6)	376 (100.0)		
Total	163 (29.2)	396 (70.8)	559 (100.0)		
**Commenced ANC**^a^**in**					
First trimester	135 (88.2)	18 (11.8)	153 (100.0)	355.9	<0.001*
Outside first trimester	28 (6.9)	378 (93.1)	406 (100.0)		
Total	163 (29.2)	396 (70.8)	559 (100.0)		

* Statistically significant

a ANC – antenatal care

FET – Fisher's Exact Test used when Chi square was inappropriate

### Follow-up and incidence of recovery from anaemia

After a median of 3.8 months (IQR: 3.2-6.2) and 1,235person-years of follow-up, the majority of patients 89.4% (n = 2,225) recovered from anaemia. One hundred and forty four (5.8%) patients were lost to follow-up and 120 (4.8%) died. The overall incidence rate of recovery from anaemia was 180 per 100 person years (95% CI: 172.9 -187.9). The period with the highest recovery rate was 0 - 3 months from initiation (1.82) and the period with the least recovery rate was > 6 months. During the 0 - 3 months from ART initiation, males had a recovery incidence rate of 237 per 100 person years (py), and females had 167 per 100 py, Table 2. During the 3-6 months follow-up time, males had recovery rates which were 2 times more than females (males: 177/100 py, females: 81/100 py).

**Table 2:** Relationship between awareness of folic acid for the prevention of birth defects and sociodemographic/maternal obstetric characteristics of the participants

Awareness of preventive role of folic acid					
Variable	Aware No (%)	Not aware No (%)	Total No (%)	χ^2^	p value
**Age (years)**					
< 30	9 (2.4)	258 (97.6)	267 (100.0)	0.188	0.664
≥ 30	8 (2.6)	284 (97.4)	292 (100.0)		
Total	17 (3.0)	542 (97.0)	559 (100.0)		
**Marital status**					
Single	1 (6.7)	14 (93.3)	15 (100.0)	0.687	0.407
Married	16 (2.9)	528 (97.1)	544 (100.0)		
Total	17 (3.0)	542 (97.0)	559 (100.0)		
**Educational status**					
University education	15 (10.9)	123 (89.1)	138 (100.0)	38.083	<0.001*
Others	2 (0.5)	419 (99.5)	421 (100.0)		
Total	17 (3.0)	542 (97.0)	559 (100.0)		
**Socioeconomic class**					
Managerial/Professional	10 (11.6)	76 (88.4)	86 (100.0)	25.593	<0.001*
Intermediate class	1 (0.9)	112 (99.1)	113 (100.0)		
Working class	6 (1.7)	354 (98.3)	360 (100.0)		
Total	17 (3.0)	542 (97.0)	559 (100.0)		
**Gravidity**					
Primigravida	8 (4.4)	175 (95.6)	183 (100.0)	1.633	0.201
Multigravida	9 (2.4)	367 (97.6)	376 (100.0)		
Total	17 (3.0)	542 (97.0)	559 (100.0)		
**Commenced ANC**^a^**in**					
First trimester	9 (5.9)	144 (94.1)	153 (100.0)	5.767	0.016*
Outside first trimester	8 (2.0)	398 (98.0)	406 (100.0)		
Total	17 (3.0)	542 (97.0)	559 (100.0)		

* Statistically significant

a ANC – antenatal care

### Predictors of recovery from anaemia

In univariate analysis, males, patients with advanced WHO clinical stage (III/IV), employed, on d4T/3TC/NVPand those on TB treatment had a higher chance of recovery from anaemia than females, patients with WHO clinical stage I/II, unemployed, on d4T/3TC/EFV and without a TB diagnosis (all p< 0.05). Higher CD4 count at ART initiation and higher BMI were associated with reduced chance of recovery from anaemia. See Table 3. The age of the patient, AZT in ART regimen, alcohol, smoking, and education status did not show any significant association with recovery from anaemia. However, in multivariate analysis only sex and WHO stage showed association with recovery from anaemia. See Table 3. In comparison to women, males had 43% higher chance of recovery from anaemia, aHR 1.43, 95%CI 1.29 -1.59. Patients with WHO clinical stage III and IV were 17% more likely to recover from anaemia compared to patients with WHO stage I and II (aHR 1.17, 95%CI 1.07-1.29). Contrary to intuitive expectation, in univariate analysis, patients initiating ART with lower CD4 count had better chance of recovery from anaemia than those with higher CD4 counts at initiation (CD4 count 50 -200cells/ml vs. CD4 count 200cells/ml vs. CD4 count

**Table 3:** Logistic regression analysis of relationship between late exposure to folic acid and sociodemographic/maternal obstetric variables

Variable	Categories	OR	95% CI	p value
**Age group**	< 30 years	1.739	0.239 – 3.650	0.144
	≥ 30 years			
**Socioeconomic class**	Working class	4.238	1.588 – 11.313	0.004*
	Intermediate class	1.159	0.481 – 2.789	0.742
	Professionals/managers			
**Educational status**	No university education	4.584	3.058 – 6.871	< 0.001*
	University education			
**Booking for ANC**	Outside 1^st^ trimester	104.267	53.094 – 204.763	< 0.001*
	Within 1^st^ trimester			
**Number of pregnancies**	Multigravida	1.995	0.951 – 4.185	0.068
	Primigravida			

* Statistically significant

## Discussion

We observed a very high proportion of patients who recovered from anaemia after initiating ART (89.4%). The majority of the patients (95%) recovered from anaemia during the first 3 months on ART. Sex and WHO clinical stage at initiation of ART were predictors of recovery from anaemia. Anaemia is a strong independent risk factor for survival [6, 7, 10, 12]. However, a significant proportion of patients already have anaemia when they initiate ART (26.5% for this cohort), and since recovery from anaemia is associated with improved survival outcomes [6, 22]this study therefore offers insight on factors that may improve survival outcomes among patients initiating ART with anaemia. The overall incidence rate of recovery from anaemia was 180 (95% CI: 172-187) per 100 person years (py). To our knowledge, we could locate any studies in sub-Saharan Africa that have looked at recovery from anaemia amongst HIV infected individuals initiating ART. On recovery from anaemia post ART initiation, most studies have looked on the average increase in haemoglobin 12 months after initiation. Our study found an average haemoglobin increase of 2.8g/dl over the first 12months post ART initiation and this is consistent with findings from other studies. Johannessen et al. (Tanzania 2011), showed that 67%(64) of anaemic participants recovered from anaemia within 12months of ART initiation and they found that on average, haemoglobin increased by 2.5 g/dl over the first 12 months [18]. A study from Uganda found that the mean haemoglobin increased by 1.5 g/dl in 12 months among patients who were anaemic at ART initiation [23].

Males had a higher chance of recovery from anaemia than females. This is in parallel with findings of previous studies that females are more likely to acquire anaemia [19, 22], possibly because of blood loss due to menstruation and multiple deliveries. We presume that thephysiological blood loss that females experience during menstruation might significantly contribute to their lower recovery rates from anaemia when compared to males. This study shows that anaemia in a female adult patient on ARTmay require more urgent attention than in a male adult patient on ART, provided other factors are held constant. In addition, with this observation it is important to understand through future studies if the prognostic value of haemoglobin level differs by gender.

Counter intuitively, participants who were more clinically ill (baseline WHO clinical stage III and IV) were 17% more likely to recover from anaemia compared to participants who were less clinically ill (baseline WHO clinical stage I and II), Table 3. Nevertheless, findings from other studies have demonstrated that WHO clinical staging is predictive of anaemia development [15, 24] and our study seems to inversely show that WHO clinical stage predicts recovery from anaemia. We speculate that because anaemic patients with advanced WHO stage (or HIV-related diseases) are likely to have anaemia of chronic diseases related to the HIV infection and would hence improve as they respond to antiretroviral therapy. On the other hand, in patients with anaemia but without advanced HIV diseases this may signify a co-morbid condition that will not be influenced by ART and needs further work up to identify i.e. malnutrition, tuberculosis or even a malignancy. We did not observe any association between baseline CD4 count and recovery from anaemia in multivariate analysis. Possibly this finding could have been different had we taken into account the longitudinal nature of CD4 count measurements rather than only a value at ART initiation.

## Conclusion

This study, demonstrated a 27% prevalence of anaemia and it showed that more than 90% of subjects recover from anaemia within 3 months after antiretroviral therapy enrolment. Predictors of recovery from anaemia are; gender and WHO clinical staging. Since anaemia is an established strong independent risk factor of mortality in HIV-infected patients, patients less likely to recover from anaemia during ART should be identified and they should receive prompt interventions to ameliorate this. Also risk stratification among patients with the same level of anaemia is important i.e. anaemia in females should signal a worse likelihood of improvement than in males. Enrolees should be taught about balanced diets as the majority of them in the cohort were underweight at initiation.

A major strength of our study is the large sample size and number of events to draw from. Additionally, we make use of routine clinical data. However, the inferences need to be interpreted with caution in light of the following study limitations. This was a retrospective study using secondary data and as such, only pre-collected information was available for analysis. Our inability to assess other important confounders which are known to have an influence on anaemia (e.g. quantify menstrual loss, co-morbid conditions and infections, use of iron supplements) may have limited this analysis. We also only looked at baseline variables so as to ascertain predictors of recovery from anaemia. Taking into account the longitudinal nature of some variables i.e. CD4 count and viral load measurements may have strengthened our findings. Also, because of the possibility of informative censoring (i.e. patients LTFU may have been the ones less likely to recover from anaemia) we may have overestimated our outcome. The TLC cohort is one of the largest urban cohorts in South Africa and is highly likely to be representative of the urban HIV infected persons but it may not be generalizable to rural HIV-infected persons who are more likely to have greater burden of anaemia.
